# Highly sensitive, self-powered and wearable electronic skin based on pressure-sensitive nanofiber woven fabric sensor

**DOI:** 10.1038/s41598-017-13281-8

**Published:** 2017-10-11

**Authors:** Yuman Zhou, Jianxin He, Hongbo Wang, Kun Qi, Nan Nan, Xiaolu You, Weili Shao, Lidan Wang, Bin Ding, Shizhong Cui

**Affiliations:** 1 School of Textile and Clothing, Jiangnan University, Wuxi, 214122 China; 2 Provincial Key Laboratory of Functional Textile Materials, Zhongyuan University of Technology, Zhengzhou, 450007 China; 3 Collaborative Innovation Center of Textile and Garment Industry, Zhengzhou, 450007 Henan China; 4 Key Laboratory of Textile Science & Technology, Ministry of Education, College of Textiles, Donghua University, Shanghai, 201620 China

## Abstract

The wearable electronic skin with high sensitivity and self-power has shown increasing prospects for applications such as human health monitoring, robotic skin, and intelligent electronic products. In this work, we introduced and demonstrated a design of highly sensitive, self-powered, and wearable electronic skin based on a pressure-sensitive nanofiber woven fabric sensor fabricated by weaving PVDF electrospun yarns of nanofibers coated with PEDOT. Particularly, the nanofiber woven fabric sensor with multi-leveled hierarchical structure, which significantly induced the change in contact area under ultra-low load, showed combined superiority of high sensitivity (18.376 kPa^−1^, at ~100 Pa), wide pressure range (0.002–10 kPa), fast response time (15 ms) and better durability (7500 cycles). More importantly, an open-circuit voltage signal of the PPNWF pressure sensor was obtained through applying periodic pressure of 10 kPa, and the output open-circuit voltage exhibited a distinct switching behavior to the applied pressure, indicating the wearable nanofiber woven fabric sensor could be self-powered under an applied pressure. Furthermore, we demonstrated the potential application of this wearable nanofiber woven fabric sensor in electronic skin for health monitoring, human motion detection, and muscle tremor detection.

## Introduction

Human skin, a natural multi-functional sensor, can transmit mechanical stimulation, temperature, humidity, and other information received from the surrounding objects to the central nervous system, and then the received information is recognized by the human brain during the neural transmission system through the inter-transformation between physical signals and chemical signals^1,2^. Pressure-sensitive electronic skins, inspired by human skin, have important applications in health monitoring, robotic skin, surgery, highly sensitive sensing equipment, and intelligent electronic products because it can mimic real human skin to detect pressure, deformation, movement, and other static or dynamic external stimuli with high accuracy^3–7^. To achieve accurate real-time health monitoring by detecting parameters such as pulse, heartbeat, and muscle vibration, electronic skin that can be applied in daily lives needs to be woven into a fabric to meet the clothing requirements of people, in addition to meeting the requirements of high sensitivity, low power and fabrication cost, and large-area implementation^8–11^.

To date, pressure-sensitive electronic skin based on flexible substrates has made great progress^12–16^. Piezoresistive sensors detect various tactile stimuli though the change of electrical resistance, and the change of contact resistance between conducting materials is one of the important approaches to realize sensors with high pressure sensitivity under mechanical deformation^17^. Currently, flexible electronic skins are usually fabricated using template-synthesis to construct multifarious structures on flexible substrates, such as interlocked micro-convex^18,19^, pyramid^20,21^, and fingerprint structures^22^, for increasing the sensitivity of the pressure sensor.

Jiang *et al*. reported a flexible nanofiber sensor with high sensitivity (15.6 kPa^−1^) and low detection limit (1.2 Pa) based on a modular assembly of a polyvinylidene fluoride-trifluoroethylene copolymer P(VDF-TrFE) nanofiber sensor encapsulated by reduced graphene oxide (rGO)^23^. Ko *et al*. prepared a piezoresistive sensor with a tunneling effect based on carbon nanotube-based composite elastomers having interlocked array structures, and they achieved a sensitivity of 15.1 kPa^−1^ at low loads^24^. Although these flexible pressure-sensitive sensors have high sensitivity, most previously reported sensors still cannot be woven for wearability owing to the limitation of their membrane structure.

Lu *et al*. proposed a simple self-assembly strategy for constructing a super-sensitive pressure sensor by coating PU yarns with ultra-thin elastic conductive layers consisting of carbon black and natural rubber, which could be woven into a fabric to monitor minute human movements^25^. Yu *et al*. fabricated an electronic fabric based on intertwined sensor electrodes with piezoresistive rubber as the shell sensing element and elastic thread coated with silver nanowires as the stretchable and highly conductive core electrode^26^. Because of the unique coaxial structure and fibrous sensor architecture, sensor had the ability to accurately capture applied loads. Despite high sensitivity and the ability to be woven, the complex yarn structures and external power supply of this wearable electronic skin remain problems to be overcome for its actual application.

Therefore, electronic skin should be self-powered to realize continuous energy supply and portability in daily use^27–30^. Dahiya *et al*. described an energy-autonomous flexible and transparent electronic skin with a novel structure, consisting of a transparent tactile sensitive layer based on single-layer graphene and a photovoltaic cell underneath as a building block for energy-autonomous^31^. This structure makes the sensor highly sensitive over a wide range of pressures (0.11–80 kPa) and had a promising alternative to replace the battery with a solar cell in the back plane of the touch sensors. Wang *et al*. introduced an electric eel-skin-inspired mechanically durable and resilient triboelectric nanogenerator by embedding AgNWs in the silicone rubber, and typical output performances of the device including open-circuit voltage, transferred charge density, and short-circuit current density were obtained by applying a 10 N contact force with different frequency^32^. These results are beneficial for a wide range of deformable power source, deformable electronics, and fully autonomous electronic skin and interactive systems.

Considering the above-mentioned problems, in the present study, we developed a design of highly sensitive, self-powered, and wearable electronic skin based on a pressure-sensitive nanofiber woven fabric sensor fabricated by weaving polyvinylidene fluoride (PVDF) electrospun yarns of nanofibers coated with poly(3,4-ethylenedioxythiophene) (PEDOT). The novel design of multi-leveled hierarchical structure for our prepared nanofiber woven fabric sensor significantly induced the change in contact area under ultra-low load, which resulted in a great change in resistance and accordingly endowed the sensor with a high pressure sensitivity. In addition, the wearable nanofiber woven fabric sensor could be self-powered with outstanding sensitivity. This wearable nanofiber woven fabric sensor has perfect application in electronic skin for health monitoring systems that can be used in daily life.

## Results and Discussion

**Figure 1:**
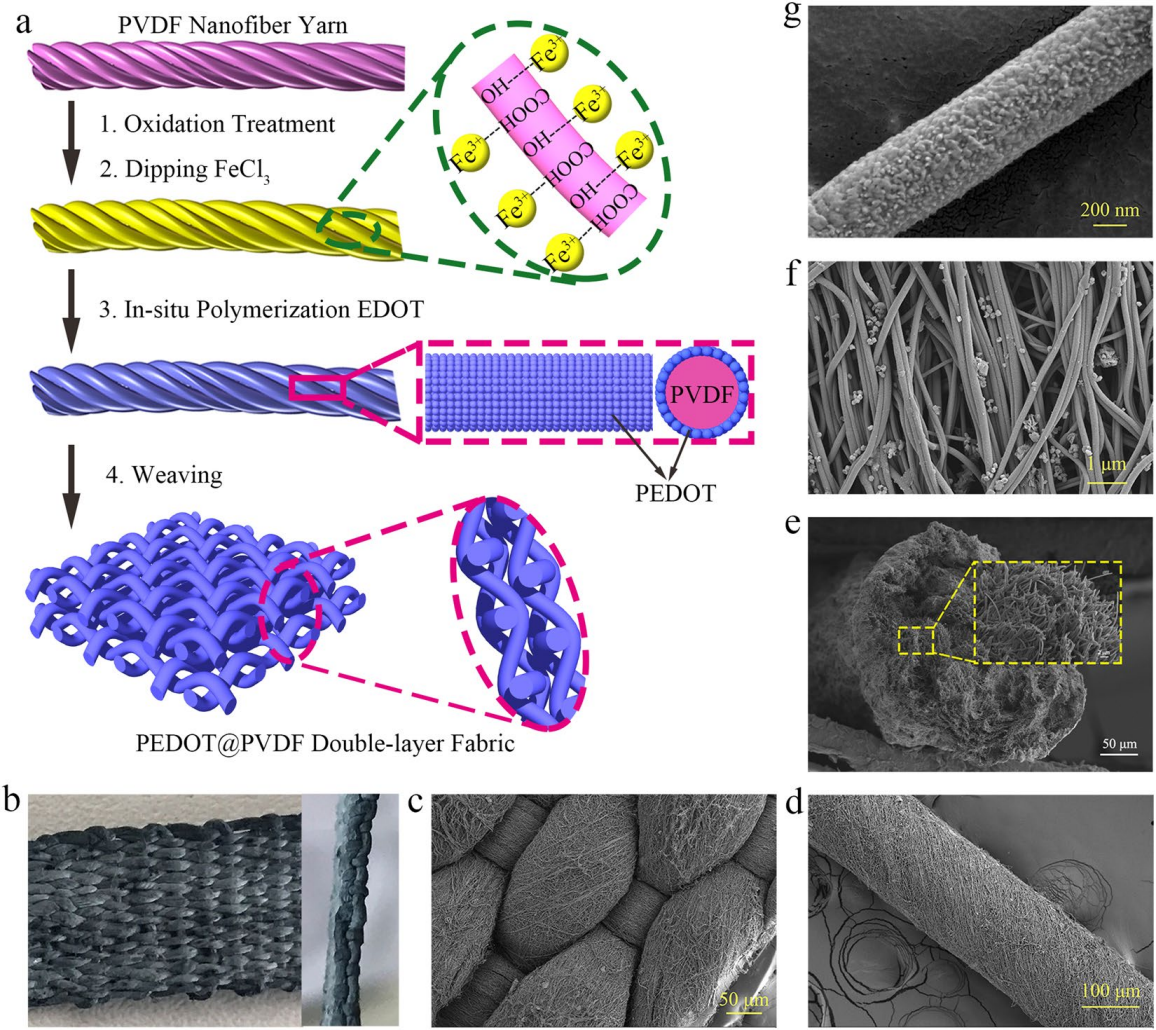
(**a**) Schematic illustrations for preparation of PEDOT@PVDF nanofiber woven fabric; (**b**) optical image of PEDOT@PVDF nanofiber fabric; SEM images of PEDOT@FVDF (**c**) nanofiber fabric, (**d**) nanofiber yarn, (**e**) oriented nanofibers, and (**f**) single nanofiber.

Figure 1a illustrates the fabrication of the PEDOT@PVDF nanofiber woven fabric. PVDF nanofiber yarns were continuously prepared using the method we previously reported^33^, and they were bundled and twisted by parallel nanofibers with diameter ranging from 100 nm to 200 nm (Figure S1). The obtained PVDF nanofiber yarns were treated with KOH/KMnO_4_ mixed solution to increase the number of oxygen-containing groups on the fiber surface. The oxidation treatment of the nanofiber yarns increased the surface roughness of the PVDF nanofiber and yielded a remarkable improvement in hydrophilicity of the yarn with a decrease of contact angle from 127° to 46° (Figure S2). The treated PVDF nanofiber yarns were dipped into a solution of FeCl_3_ in ethanol, and the adsorption of FeCl_3_ as a catalyst for the polymerization of EDOT on the fiber surface transformed the color of the yarn from white to light yellow. The resulting PVDF nanofiber yarns were immersed in a solution of EDOT in chloroform, and EDOT was polymerized *in situ* on the surface of the fibers in the yarn, with FeCl_3_ acting as a catalyst, to form PEDOT@PVDF nanofiber yarns composed of PEDOT-coated PVDF nanofibers. After the *in-situ* polymerization of EDOT on the fiber surface, the color of the PEDOT@PVDF nanofiber yarn changed to blue-green. It should also be noted that the stress intensity of PEDOT@PVDF nanofibers yarns is close to that of commercial viscose filament, and were able to meet the requirement of weaving (Figure S3). The obtained PEDOT@PVDF nanofiber yarns could be easily woven into a double-layer fabric, following which a wearable PEDOT@PVDF nanofiber woven fabric (PPNWF) pressure sensor with a sandwich structure was assembled by gluing PDMS films with copper wires on both the upper and lower sides of the double-layer fabric (Figure S4).

**Figure 2:**
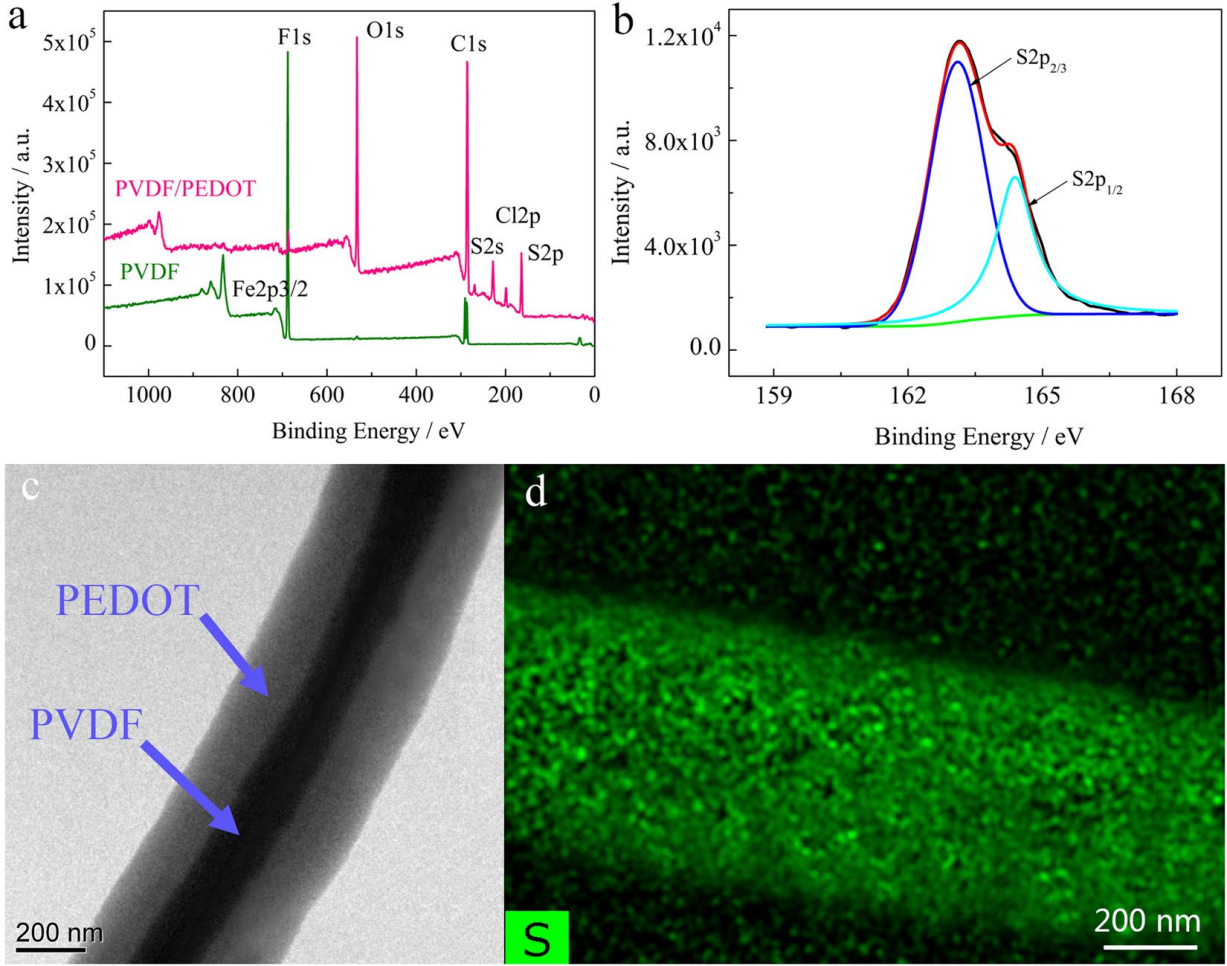
(**a**) TEM image of PEDOT@PVDF nanofiber; (**b**) mapping image of the S element in PEDOT; XPS results of (**c**) wide-scan spectrum of PVDF and PEDOT@PVDF nanofiber yarn and (**d**) S2p spectrum of PEDOT in PEDOT@PVDF nanofiber yarn.

Figure 1b shows an optical image of the PPNWF. A perfect weaving structure was observed in typical scanning electron microscopy (SEM) images of the PEDOT@PVDF nanofiber fabric, which the warp and weft yarns are crisscross assembled and interlaced with each other in an over-and-under fashion (Fig. 1c). Subsequently, a multi-joint channel system is formed in which the interlacing of warp and weft yarns formed a regular protuberance acting as the mechanical sensing unit. The double-layer fabric structure enhanced the interlocking possibility of regular sites because external pressure induces stress to focus on the small sites and deforms the sites. Figure 1d and e shows typical SEM images of the PEDOT@PVDF nanofiber yarn, in which the fibers are almost arranged in parallel in the direction of twist with its diameter increasing from the range of 100–200 nm to 500–600 nm after PEDOT coating (Fig. 1f). The enlarged field-emission SEM (FESEM) image of a single PEDOT@PVDF nanofiber in Fig. 1g revealed an accidented topography on the fiber surface, and the fiber was evenly coated by *in-situ* polymerized PEDOT particles with size in the range of 30–50 nm. X-ray photoelectron spectroscopy (XPS) spectra of PEDOT@PVDF nanofiber yarn demonstrated the disappearance of the characteristic F-signal peaks of PVDF and the appearance of O- and S-signal peaks (Fig. 2a). Moreover, the XPS S2p de-convolution spectra of PEDOT@PVDF nanofiber yarn showed peaks located at 163.4 eV and 164.8 eV, corresponding to the binding energy of S2p3/2 and S2p1/2 of PEDOT, respectively (Fig. 2b), which is consistent with previous reports^34–36^. The transmission electron microscopy (TEM) image shown in Fig. 2c also confirmed the core-shell structure of the nanofiber in the PEDOT@PVDF nanofiber yarn with a continuously coated PEDOT shell layer, and the mapping image of S element in PEDOT revealed S element was distributed uniformly on the fiber surface (Fig. 2d). The above results demonstrate that the PVDF nanofibers were uniformly coated with PEDOT in yarns, which could be used as continuous self-assembled conductive pathways in the PPNWF pressure sensor.

Hierarchical geometrical features inspired by structures found in nature have displayed many advantages, one of which is the maximized contact area^37^. Cho *et al*. applied three-level hierarchically structured Gr/PDMS arrays with protuberances having a diameter of 59 μm and microdomes having a diameter of 3.2 μm to pressure-sensitive electronic skin and demonstrated a high sensitivity of 8.5 kPa^−1^ at low loads and a wide pressure range (~12 kPa)^38^. Similarly, the interlaced joint of warp and weft yarns as a protuberance at a dimension of 200–300 μm was composed of submicro-scale fibers 500–600 nm in size coated continuously with nanoscale PEDOT nanoparticles 30–50 nm in size. The multi-level dome arrays from the microscale to nanoscale in the PPNWF pressure sensor can be modeled as a hierarchical structure, which can afford more contact joints and a large variation in contact area for pressure sensing, thereby contributing to the ability to capture subtle stresses and to the increase of sensitivity.

The XRD pattern in Figure S4c shows a transformation in the crystalline texture of PVDF powder from the α-phase to β-phase after electrospinning, and only the β-phase of PVDF showed piezoelectricity^39,40^, which was well maintained after the *in-situ* polymerization of EDOT coating.

**Figure 3:**
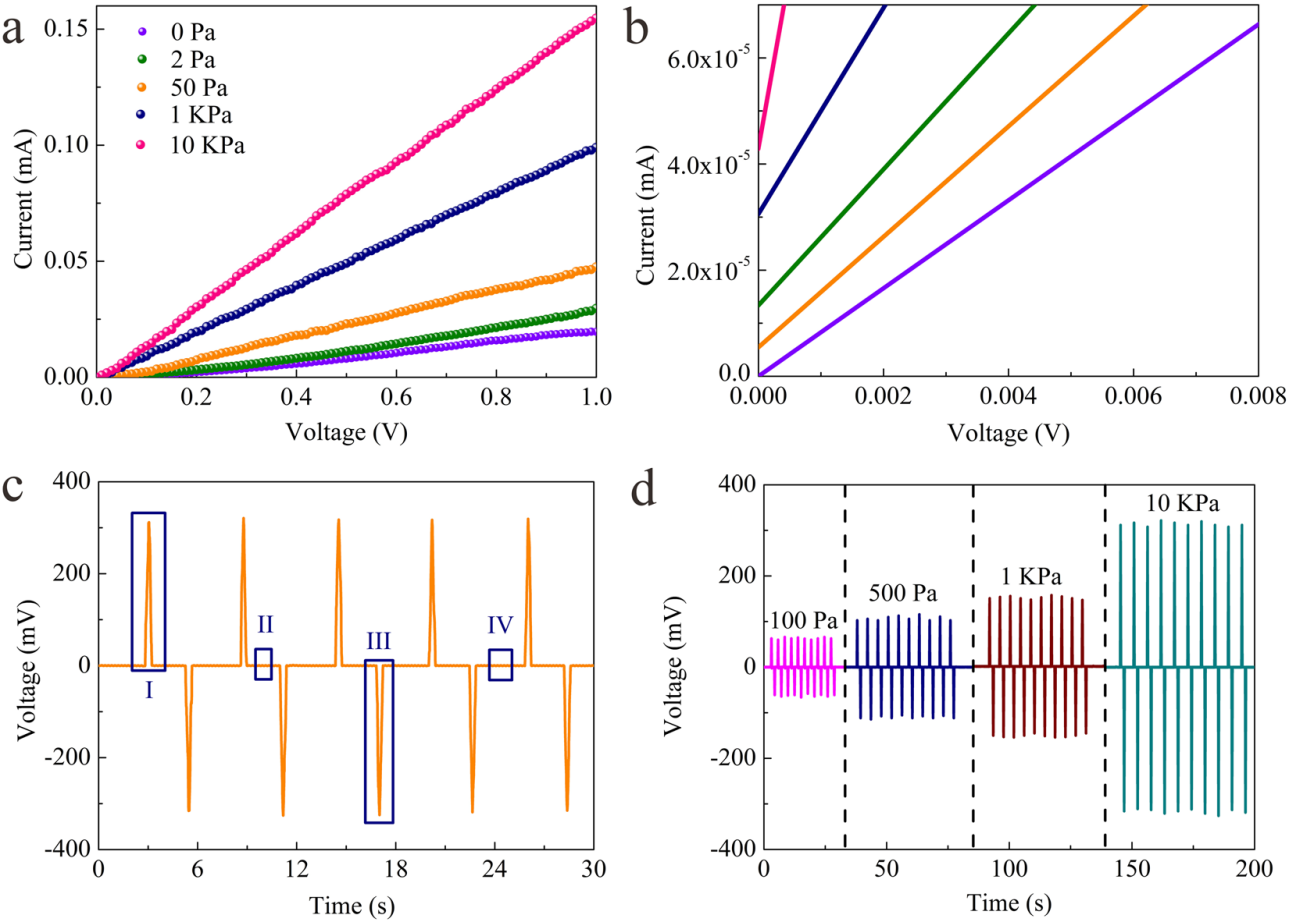
(**a**) I-V curves of PPNWF pressure sensor and (**b**) initial current under different pressures; (**c**) open-circuit voltage signal of PPNWF pressure sensor under an applied periodic pressure of 10 kPa; (**d**) voltage signal of PPNWF pressure sensor under different pressures.

A self-made pressure-sensitive test system was assembled to study the pressure sensitivity of the PPNWF pressure sensor (Figure S5). The current-voltage (I-V) curve of the PPNWF pressure sensor showed that the current of our fabricated sensor, which was used as a resistive element in this testing circuit of volt-ampere characteristic, varied linearly with the voltage (Fig. 3a), indicating the sensor had good ohmic behavior with a stable response to pressure and the current increased significantly with pressure increasing from 0 Pa to 10 kPa. During the testing process of current-voltage characteristic, the working voltage is changed from 0 V to 1 V. Theoretically, the current of the sensor should be 0 A at the origin when the initial working voltage is 0 V, regardless of how much the resistance of the PPNWF pressure sensor changed under the pressure. Noticeably, an initial current existed at the origin when different pressures were applied to the sensor, and the initial current of the sensor increased from 0 mA to 4.328 × 10^–5^ mA when the pressure loading was increased from 0 Pa to 10 kPa. (Fig. 3b). This phenomenon indicated that, there had another voltage in the circuit in addition to the testing voltage under increasing pressure, which led to the existence of the current. In other word, our sensor might be self-powered. In order to eliminate the interference of external factors, we used a piezoelectric performance testing system to test the self-powering effect of the PPNWF pressure sensor. We obtained an open-circuit voltage signal of the PPNWF pressure sensor under an applied periodic pressure of 10 kPa through the piezoelectric performance testing system, and the output voltage exhibited a distinct switching behavior to the applied pressure (Fig. 3c). The reason of the self-powering effect for the PPNWF pressure sensor could be explained by the piezoelectricity of PVDF β-phase. Furthermore, the voltage generated in the PPNWF pressure sensor increased with the increase of applied pressure (Fig. 3d).

**Figure 4:**
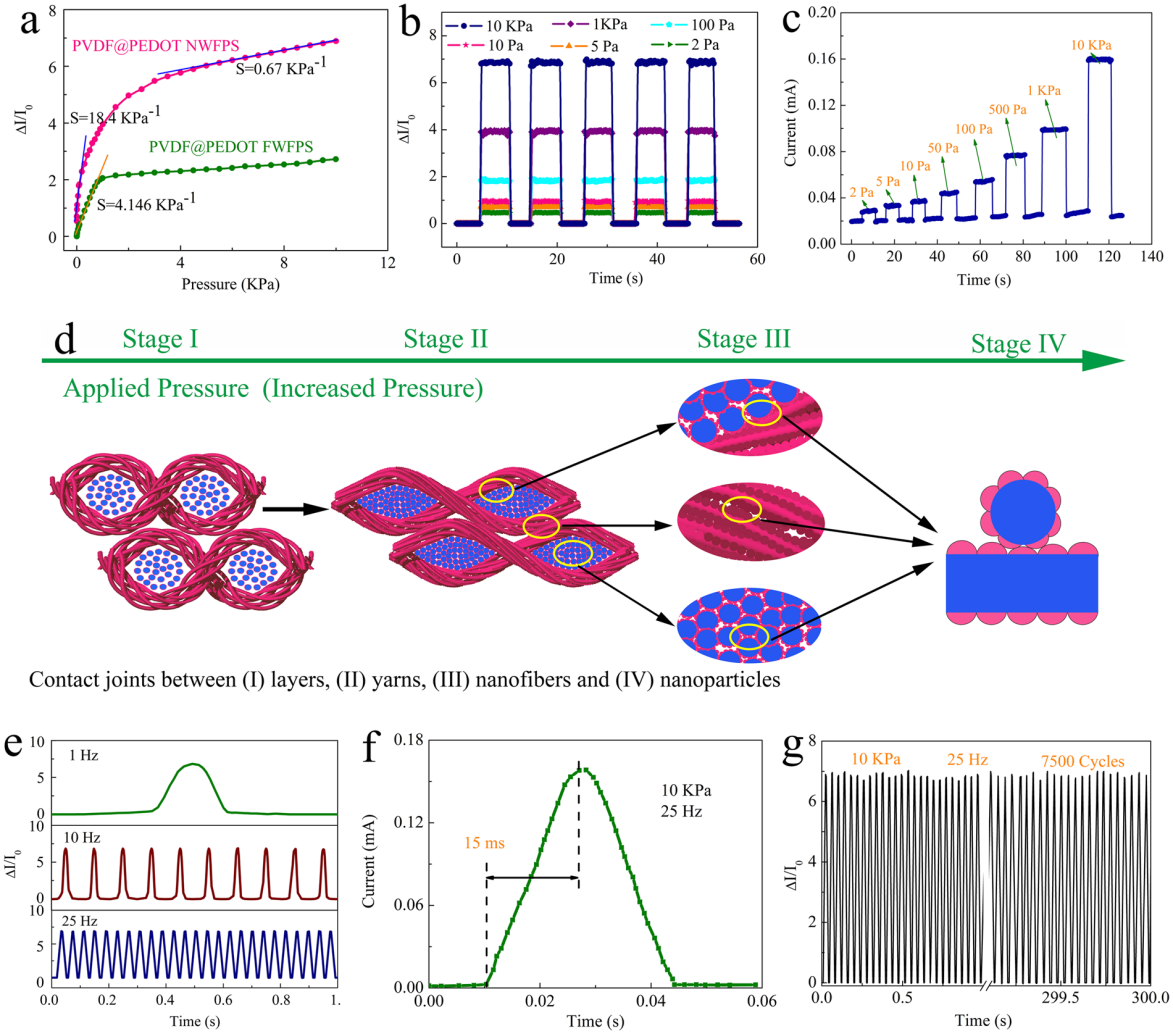
(**a**) Relative change in current versus applied pressure; (**b**) Static current response under varying pressure; (**c**) Dynamic instantaneous current response to varying pressure; (**d**) Schematic diagram of the main structural change in the PPNWF pressure sensor during the compression process; (**e**) Response to applied pressure with various frequencies; (**f**) a typical signal of current change at 25 Hz in (**e**); (**g**) Durability recorded by repeated loading-unloading cycles at a frequency of 25 Hz and a pressure of 10 kPa.

The pressure sensitivity of the PPNWF pressure sensor was further investigated by monitoring the relative current change under different pressure loads (${\rm{\Delta }}I/{I}_{0}$) at a working voltage of 1 V. The pressure sensitivity S of the pressure sensor is defined as the slope of the curve shown in Fig. 4a. The pressure sensitivity S is calculated as follows:

1 $$S=({\rm{\Delta }}I/{I}_{0})/{\rm{\Delta }}P$$

where ${\rm{\Delta }}I$ is the pressure-induced change in current (mA), ${I}_{0}$ is the initial current of the sensor without pressure loading (mA), and ${\rm{\Delta }}P$ is the change in applied pressure (kPa).

As shown in Fig. 4a, the relative change in current of the prepared PPNWF pressure sensor against applied pressure exhibited a linear region at a relatively low pressure range (~100 Pa) with sensitivity up to 18.4 kPa^−1^, and the sensitivity decreased to 0.7 kPa^−1^ at a relatively high pressure range. To demonstrate the ultra-high sensitivity of the PPNWF pressure sensor, a PEDOT-coated PVDF filament woven fabric sensor was fabricated for comparison using common filaments with diameter similar to that of the PVDF nanofiber yarns and under the same process of PEDOT polymerized coating (Figure S6). The PEDOT-coated PVDF filament woven fabric sensor showed a lower sensitivity of 4.146 kPa^−1^ at a pressure of ~100 Pa. We attributed the enhanced sensitivity of the PPNWF pressure sensor to the drastic change in contact area under low pressure, which is derived from its multi-level hierarchical structure (Fig. 4d). The accumulation of nanofibers, which were much higher in number compared to the filaments, deformed easily with massive protuberances of PEDOT nanoparticles, resulting in a significant increase of contact joints and the total contact area under subtle stress; thus, our prepared sensor had an ultra-high sensitivity. On the other hand, the change of contact area in the PEDOT-coated PVDF filament woven fabric sensor under pressure only depended on the contact deformation of filaments, and there was no additional submicro- or nano-structure to be deformed by applied pressure (Figure S7). The decreased sensitivity of the PPNWF pressure sensor under a pressure loading greater than 2 kPa was mainly caused by the increase in conductivity of PEDOT@PVDF nanofibers due to the nearly stable contact area under such a large pressure, which required an excessively large load to make PEDOT nanoparticles closely contact the fiber.

Therefore, the PPNWF pressure sensor had both changes in resistance and voltage under applied pressure. Simultaneous changes in voltage and resistance caused the PPNWF pressure sensor to have pressure-sensitivity, so that the sensor was in addition to be piezoresistive also piezoelectric. The total changes in current of the prepared PPNWF pressure sensor under applied pressure was shown in equation (2). It should be noted that, although applied pressure could lead to the changes in voltage and resistance at the same time, the two were not affected each other because of the different change mechanisms. Moreover, the pressure-sensitivity caused by piezoelectricity was poor compared to the piezoresistive pressure-sensitivity because the change in voltage was lower than in resistance. The sensor could generate a weak open-circuit voltage signal under applied pressure, while had a large change in resistance owing to the multi-level hierarchical structure of the sensor. Therefore, the high pressure-sensitivity of the sensor prepared in this research was mainly caused by the change in resistance.

2 $$\frac{{\rm{\Delta }}I}{{I}_{0}}=\frac{\frac{{U}_{0}+U^{\prime} }{{R}_{0}-R^{\prime} }-\frac{{U}_{0}}{{R}_{0}}}{\frac{{U}_{0}}{{R}_{0}}}=\frac{1+\frac{U^{\prime} }{{U}_{0}}}{1-\frac{R^{\prime} }{{R}_{0}}}$$

where ${U}_{0}$ is the working voltage of 1 V, $U^{\prime}$ is the open-circuit voltage generated by the sensor under applied pressure (mV), ${R}_{0}$ is the initial resistance of the sensor without pressure loading (Ω), and $R^{\prime}$ is the resistance under applied pressure (Ω).

Static pressures ranging from 2 Pa to 10 kPa were applied to study the static pressure response of the PPNWF pressure sensors at a working voltage of 1 V. As shown in Fig. 4b, a significant response in terms of current change was observed for the PPNWF pressure sensor under an ultra-low pressure (2–5 Pa) even under an applied voltage of 1 V, indicating that our sensor had an ultra-low pressure-detection limit. Meanwhile, the PPNWF pressure sensor also showed stable pressure sensitivity over a wide pressure range. It is obvious that the increase of applied static pressure would induce a greater change in current and, thereby, a greater pressure response. In addition, Fig. 4c shows the dynamic instantaneous current response of the PPNWF pressure sensor to varying pressure (ranging from 2 Pa to 10 kPa) under a constant working voltage of 1 V, and the response values of the current corresponded to the variation in pressure. The current almost immediately responded to a sudden external pressure and was instantly restored to the initial value at any pressure once the external pressure was removed, demonstrating the excellent response sensitivity of our sensor.

The response speed and durability, which are two important parameters for PPNWF pressure sensors, were further tested. The results of response speed for the PPNWF pressure sensor, which was tested by applying impulse pressures of 1 Hz, 10 Hz, and 25 Hz, showed that the sensor could work stably over a wide range of frequency (Fig. 4e). The PPNWF pressure sensor showed an instantaneous response with a speed of 15 ms when the frequency of applied impulse pressure was 25 Hz, which could be attributed to its unique multi-level hierarchical structure (Fig. 4f). However, the response time of the PPNWF pressure sensor increased as the frequency of applied pressure decreased, which was mainly due to a decrease of the speed for applying pressure. Furthermore, the durability of the PPNWF pressure sensor was verified by repeating loading-unloading cycles at a frequency of 25 Hz and a pressure of 10 kPa (Fig. 4g). The variation in current remained at the same level during the process of repeated loading-unloading for 7500 cycles, indicating that our sensor had excellent cycling stability and repeatability.

**Figure 5:**
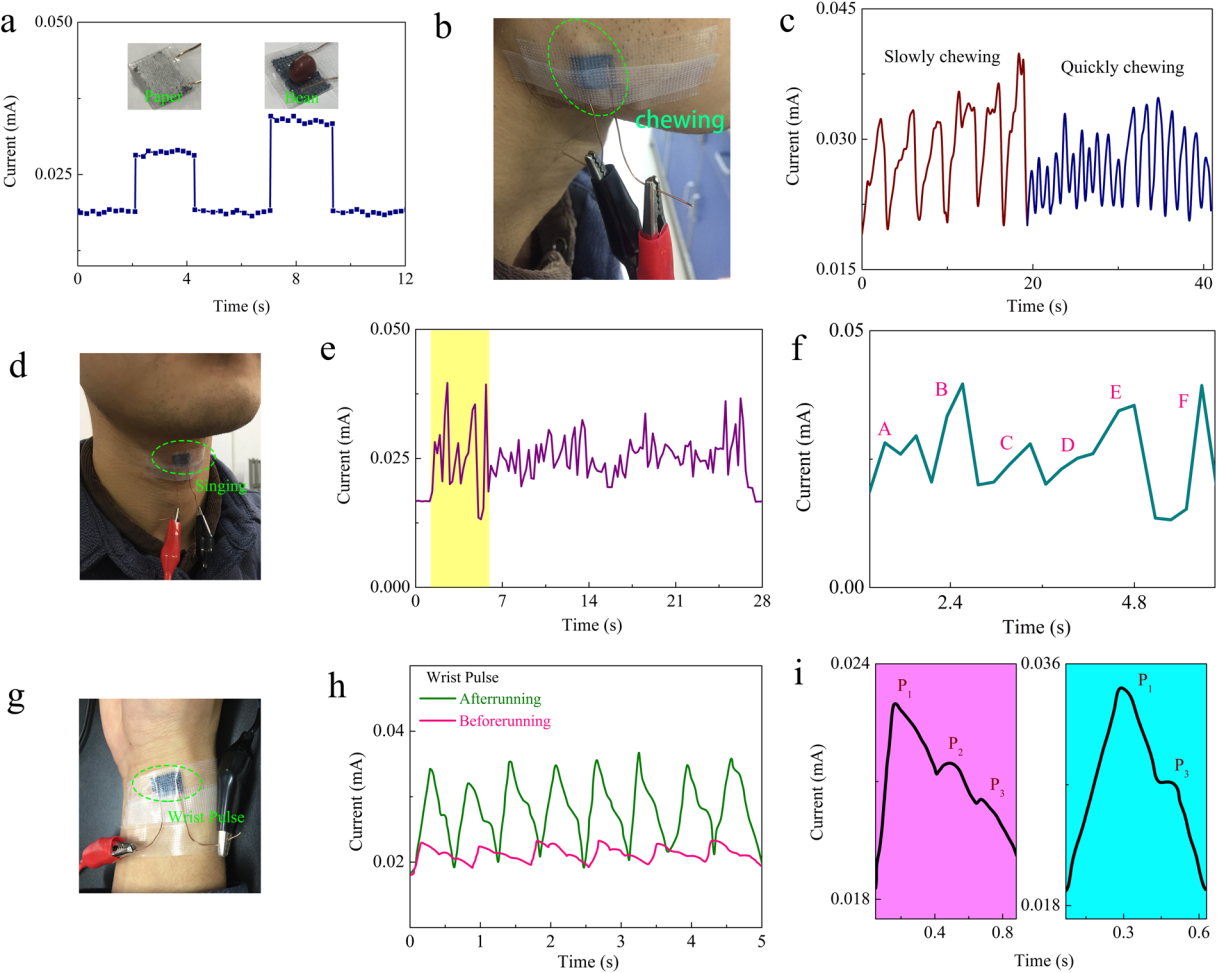
Application of PPNWF pressure sensor. (**a**) current response to ultra-light objects; (**b**,**c**) monitoring of muscle movement on human face; (**d**–**f**) monitoring of voice when people sang; (**g**–**i**) current change according to wrist pulse before and after exercise.

Our PPNWF pressure sensor could be successfully used as wearable electronic skin to detect subtle stresses sensitively and has outstanding potential for application in human health monitoring and small-scale movement detection. For demonstration, an ultra-light object, such as a piece of paper (15 mg) or a grain of beans (50 mg), was placed on the surface of the PPNWF pressure sensor, and a corresponding response in terms of an increased current was instantaneously generated (Fig. 5a). Moreover, we attached the PPNWF pressure sensor on the masseter portion of the human face to monitor muscle movement (Fig. 5b). When the subject bit teeth repeatedly, the PPNWF pressure sensor instantaneously generated a synchronized current response due to the movement of the facial masseter (Fig. 5c). If the subject slowly bit teeth, the PPNWF pressure sensor generated a slowly increasing current response because the masseter imposed a slowly increasing pressure on the sensor. However, the frequency of current response observably increased with a decreased current value when the subject bit teeth rapidly because the force imposed by the masseter decreased as the frequency increased (Movie 1). When our PPNWF pressure sensor was attached to a human throat, a significant change in current was observed as the subject sang ABC songs (Fig. 5d,e), and the tone could be distinguished according to the value of current change (Fig. 5f; Movie 2). A PPNWF pressure sensor attached to a human wrist could successfully monitor the pulse (Fig. 5g). Figure 5h shows the current change of the PPNWF pressure sensor attached to the wrist of a healthy male subject (age: 22 years, height: 178 cm, weight: 67 kg) before and after exercise. The recorded results clearly showed the pulse frequency, pulse shape, and repeatability of single pulse peaks for the subject before and after exercise. After exercise, the intensity and frequency of pulse increased significantly, and the typical percussion, tidal, and diastolic peak marked as P1, P2, and P3, respectively, in a single pulse waveform could be observed clearly (Fig. 5i). The sensor we prepared can monitor the wrist pulse, record the three typical peaks of P1, P2 and P3 in the single pulse of healthy human body with tranquility, and even distinguish the change of the pulse frequency and pulse shape for the subject before and after exercise. When the human body have potential issues or disease, such as heart disease and hypertension, its pulse must be different from the healthy body. We can use our sensor with low-pressure detection to monitor the pulse, and easily infer whether there have potential issues or disease for the human body through observing the changes of the waveform, frequency and intensity for the pulse, which could reduce unnecessary trouble. It also can be explained that our sensor has a potential application as electronic skin for human health monitoring. In summary, the PPNWF pressure sensor has the ability to detect various pressures, including ultra-low pressures, and the fabric is flexible and wearable. Thus, the sensor has a good potential for application in wearable health monitoring and the detection of human body movement. It is worth noting that the proposed method also provides an idea for the fabrication of functional electronic textiles.

## Experimental Section

### Fabrication of PVDF nanofiber yarn

PVDF nanofiber yarns were continuously prepared using the method we previously reported^33^. PVDF solution (16.5 wt%) was transported at a uniform rate through liquid-transport tubes to two sets of nozzles arranged symmetrically via fluid-supply apparatus (Figure S8). The nanofibers were electrospun by electric-field force and deposited in a rotary funnel to form a nanofiber web that covered the end of the funnel based on the conjugate principle of electrospinning, following which it was pulled into fiber bundles. Finally, the nanofiber yarn was obtained by twisting the fiber bundles through the rotary funnel and continuously winding the bundles with a yarn winder (Figure S9).

### ***In-situ*** polymerization of EDOT on PVDF nanofiber surface

The obtained PVDF nanofiber yarns were first dipped into absolute ethanol for 24 h and then into deionized water for 30 min to displace the residual ethanol. The ethanol-treated yarns without dripping were first immersed in a mixed solution of potassium hydroxide (KOH, 2.5 mol/L) and potassium permanganate (KMnO4, 3 wt%) for 10 min, then in sodium bisulfite (NaHSO3, 2 wt%) solution until the yarn color became white, subsequently in hydrogen peroxide solution for 10 min, and finally placed in an oven for drying at 60 °C. The modified PVDF nanofiber yarns were immersed in a solution of FeCl_3_ in ethanol for 10 min and then dried at 60 °C. In the end, the PVDF nanofiber yarns soaked in FeCl_3_ oxidant were immersed in a solution of EDOT in chloroform for 20 h, then washed repeatedly with ethanol, and dried again at 60 °C to obtain PEDOT@PVDF nanofiber yarns.

### Assembly of PPNWF pressure sensor

The obtained PEDOT@PVDF nanofiber yarns were used as warp and weft yarns for crisscross assembling and interlacing with each other in an over-and-under fashion for weaving into a double-layer fabric. Subsequently, a wearable nanofiber woven fabric sensor with a sandwich structure was assembled by adhering PDMS films (curing agent-to-base ratio of 1:10) with copper wires on both the upper and lower sides of the double-layer fabric. During the gradual curing of the PDMS film, the fabric with a wire on its surface was embed in the almost curing PDMS film. The wire was tightly attached to the fabric surface by the bonding of the PDMS film surface and the fabric surface.

### Characterization

Field-emission scanning electron microscopy (FESEM; Hitachi S-4800, Japan) was used to examine the morphologies of the nanofiber fabrics, nanofiber yarns, and nanofibers. Transmission electron microscopy (TEM; accelerating voltage = 20 kV, Hitachi H-800, Japan) was used to study the interior structure of the PEDOT@PVDF nanofibers. X-ray photoelectron spectroscopy (XPS; Thermo Scientific ESCALAB-250, USA) with non-monochromatized Cu Kα X-rays applied as the excitation source was used to characterize the composition of the PEDOT@PVDF nanofiber. X-ray diffraction (XRD, Rigaku D-max 2200, Japan) was used to characterize the crystal structure of the PVDF power, PVDF nanofiber, and PEDOT@PVDF nanofiber by using Cu Kα radiation (λ = 0.15406 nm) applied over a 2θ range of 10°-70° at a scanning speed of 5°/min. The contact angle of the specimens was measured using a Dataphysics OCA20 (Germany) tester at room temperature and humidity. The stress intensity was measured with an INSTRON 365 tester, and the electrical response of the sensor was recorded in real time by using a Keithley 4200-SCS digital meter with a test step of 10 ms. The piezoelectric performance was recorded by using a voltage amplifier and a dynamic signal test and analysis system (DH5922N).

## Conclusion

We developed a simple and effective method for the preparation of a flexible, self-powered, and wearable nanofiber fabric sensor by interweaving PEDOT@PVDF nanofiber yarns composed of PEDOT-coated PVDF nanofibers. The special multi-level hierarchical structure of the PPNWF pressure sensor yielded a high sensitivity (18.376 kPa^−1^ at ~100 Pa), wide pressure range (0.002–10 kPa), fast response (15 ms), and high durability (7500 cycles). In addition, the PPNWF pressure sensor could be self-powered because of the piezoelectricity of PVDF β-phase, and the output voltage exhibited a distinct switching behavior to applied pressure. We also demonstrated the use of the proposed sensor as electronic skin for monitoring subtle human motion, muscle vibration, and health in daily life. We believe our study will contribute effectually toward the development of advanced wearable pressure sensors that have potential applications as electronic skin for human health monitoring, human-machine interfaces, and biomedical prostheses.

## Electronic supplementary material

Supplementary Information

Movie S1

Movie S1
