# Supplementary material for: Highly sensitive, self-powered and wearable electronic skin based on pressure-sensitive nanofiber woven fabric sensor
Source: Sci Rep. 2017 Oct 11;7:12949. doi: 10.1038/s41598-017-13281-8 (PMC5636882; doi:10.1038/s41598-017-13281-8)
Supplement: Supplementary file 1 — Supplementary Information [file 41598_2017_13281_MOESM1_ESM.doc]

**SUPPORTING INFORNATION**

**Highly sensitive, self-powered and wearable electronic skin based on pressure-sensitive nanofiber woven fabric sensor**

Yuman Zhou^a^, Jianxin He^b,c^*******, Hongbo Wang^a^*******, Kun Qi^a^, Nan Nan^b,c^, Xiaolu You^b,c^, Weili Shao^b,c^, Lidan Wang^b,c^, Bin Ding^b,d^, Shizhong Cui^b,c^

^a^ School of Textile and Clothing, Jiangnan University, Wuxi 214122, China

^b^ Provincial Key Laboratory of Functional Textile Materials, Zhongyuan University of Technology, Zhengzhou 450007, China

^c^ Collaborative Innovation Center of Textile and Garment Industry, Zhengzhou 450007, China Henan

^d^ Key Laboratory of Textile Science & Technology, Ministry of Education, College of Textiles, Donghua University, Shanghai 201620, China

**Corresponding author:** Jianxin He

P.O. Box 110, College of Textiles, Zhongyuan University of Technology, 41 Zhongyuan Road, Zhengzhou City 450007, Henan Province, People’s Republic of China

**E-mail:** [hejianxin771117@163.com](mailto:hejianxin771117@163.com)

**Co-corresponding author:** Hongbo Wang

School of Textile and Clothing, Jiangnan University, 1800 Lihu Road, Wuxi City 214122, Jiangsu Province, People’s Republic of China

**E-mail:** wxwanghb@163.com


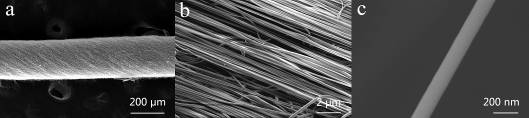


Figure S1 SEM images of (a) PVDF nanofiber yarn, (b) oriented nanofibers, and (c) single nanofiber.


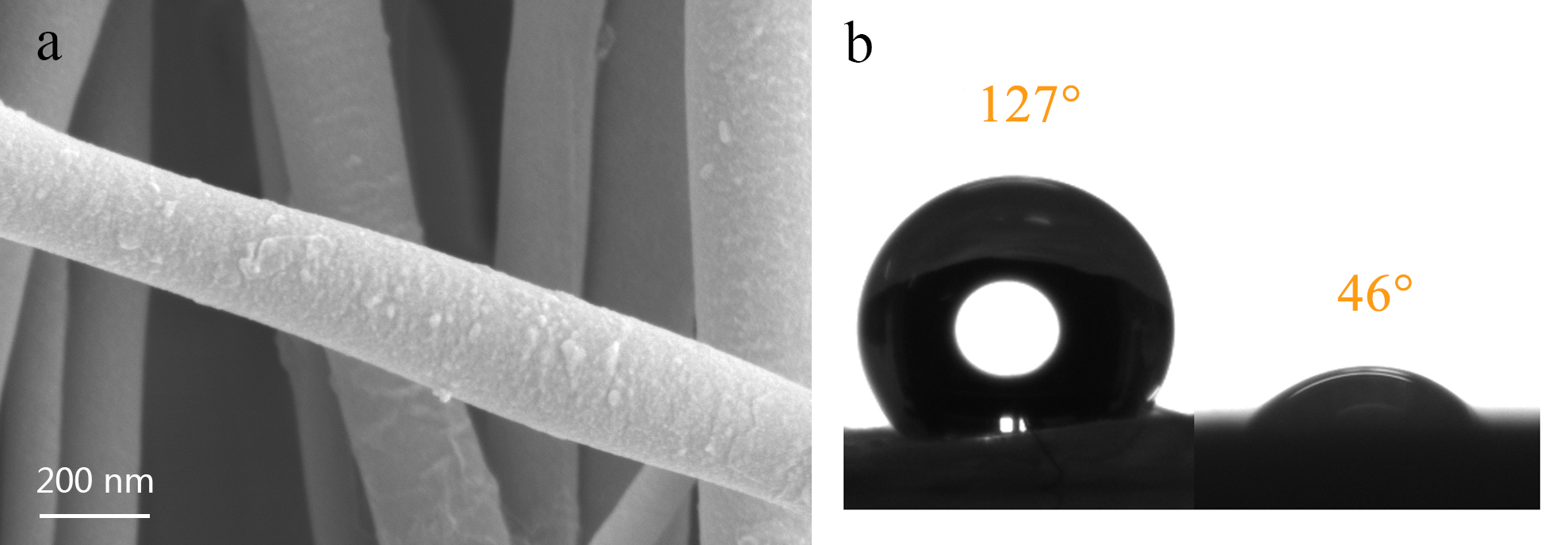


Figure S2 (a) SEM image of PVDF nanofibers and (b) contact angle of PVDF nanofiber after oxidation treatment.


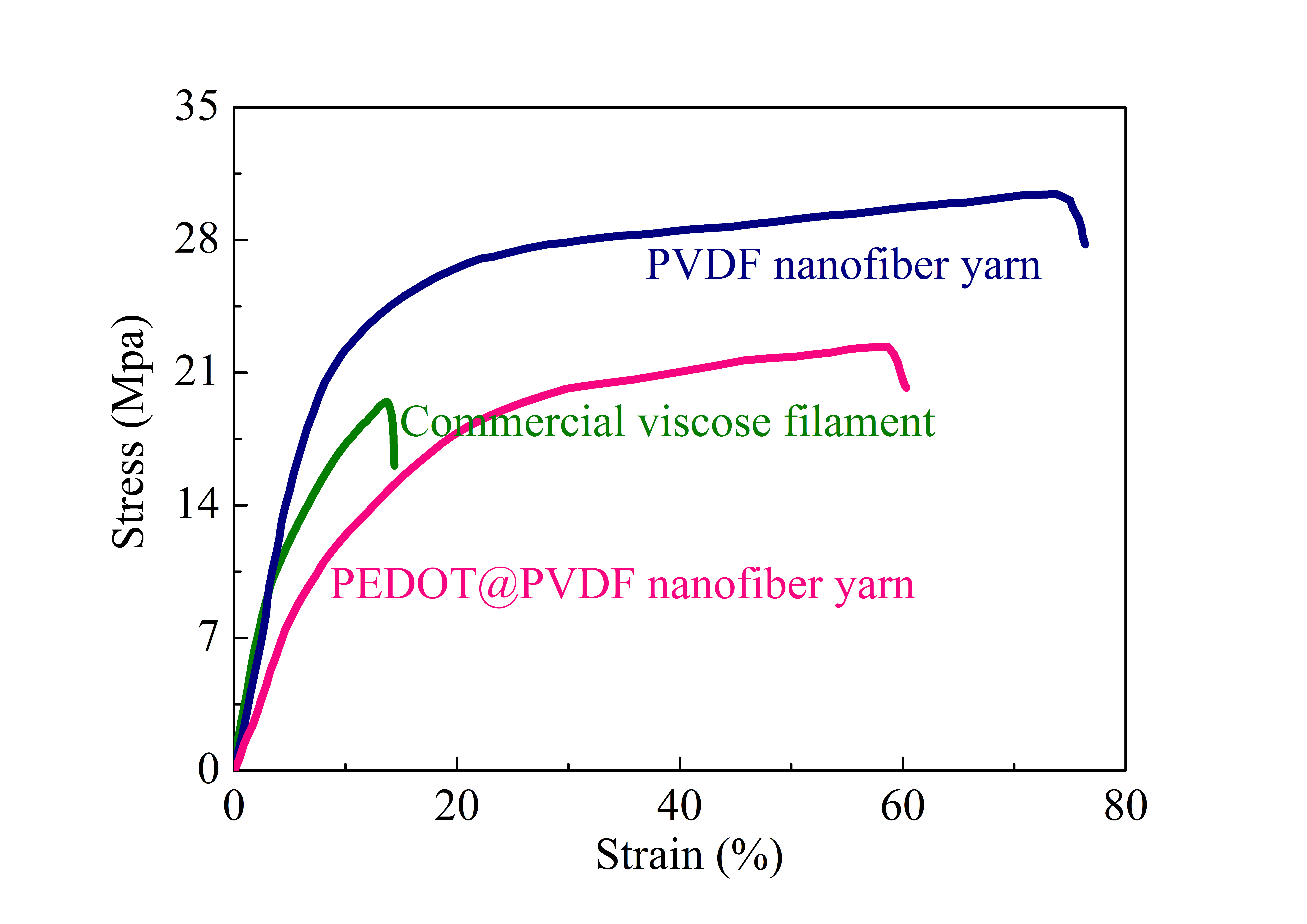


Figure S3 Stress intensity of PVDF nanofiber yarn, PEDOT@PVDF nanofiber yarn, and commercial viscose filament.


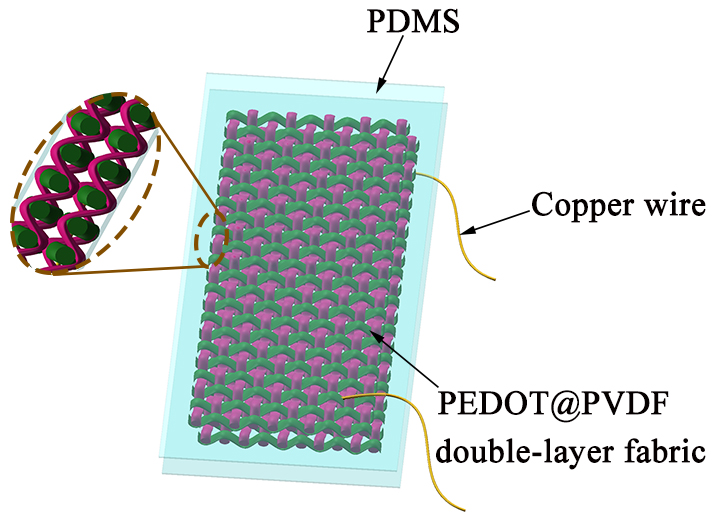


Figure S4 The schematic diagram of the PPNWF pressure sensor


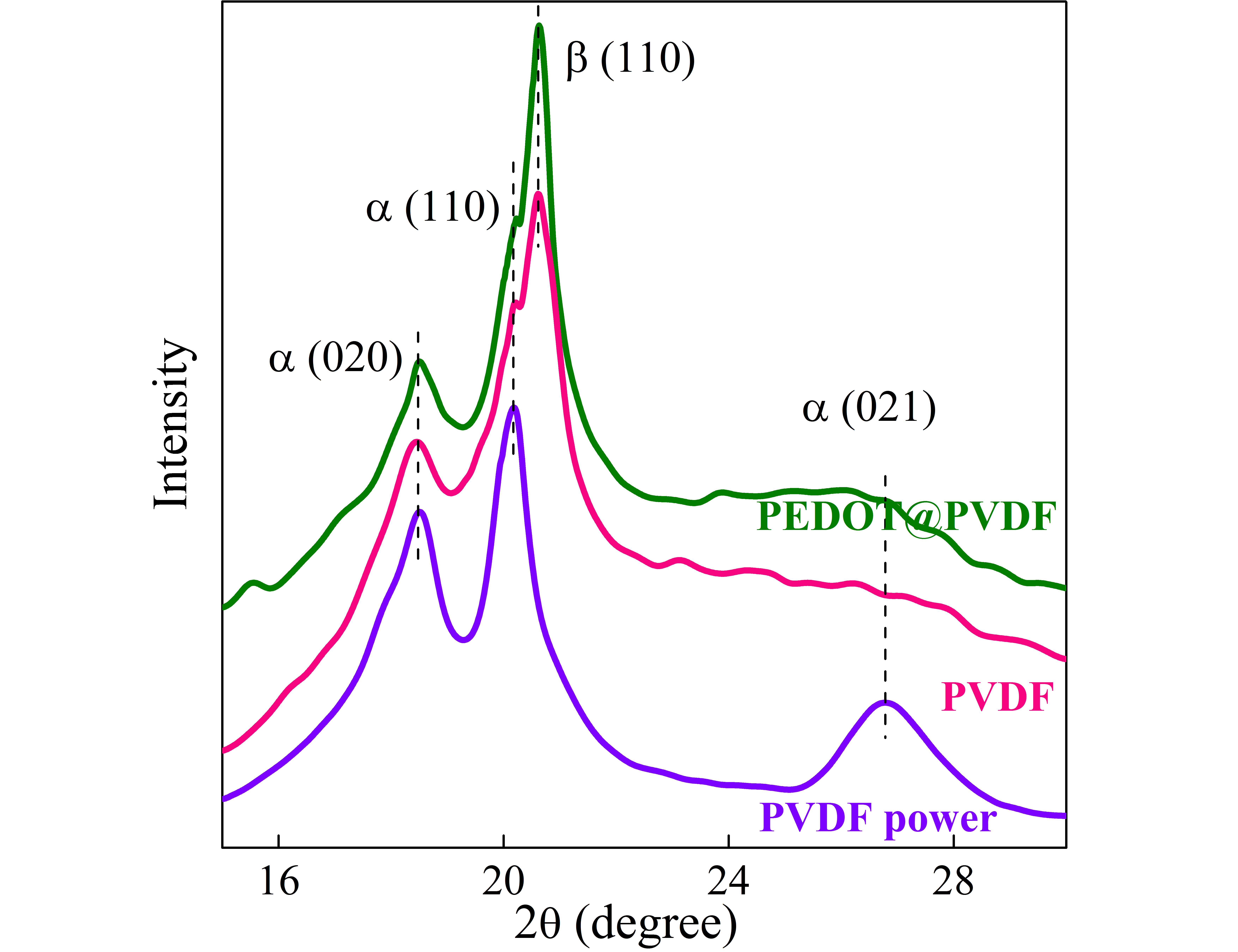


Figure S5 XRD patterns of PVDF power, PVDF nanofiber yarn and PEDOT@PVDF nanofiber yarn.


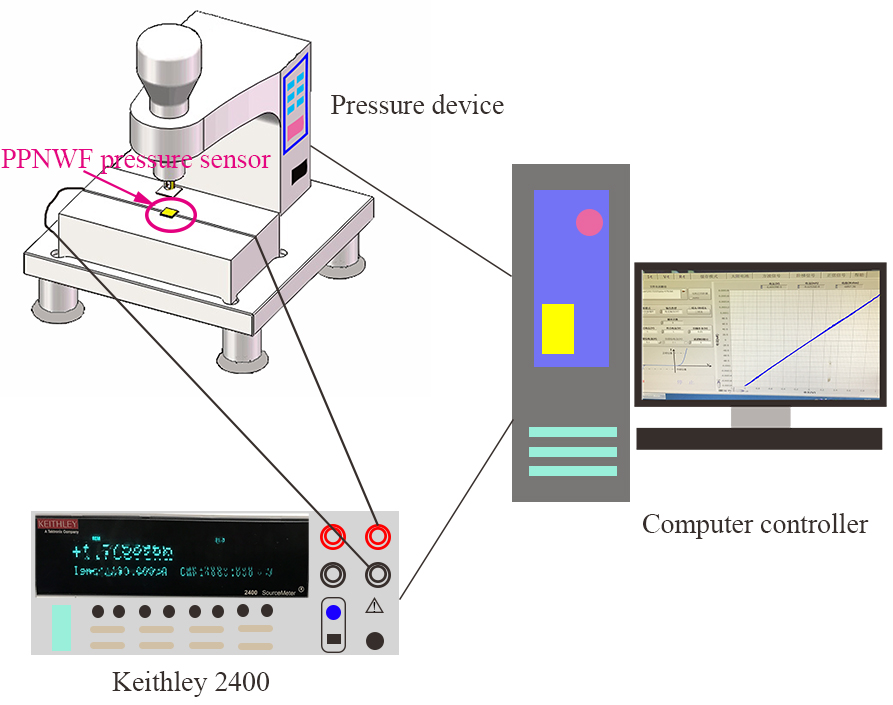


Figure S6 Schematic of the self-made test system for pressure sensitivity.


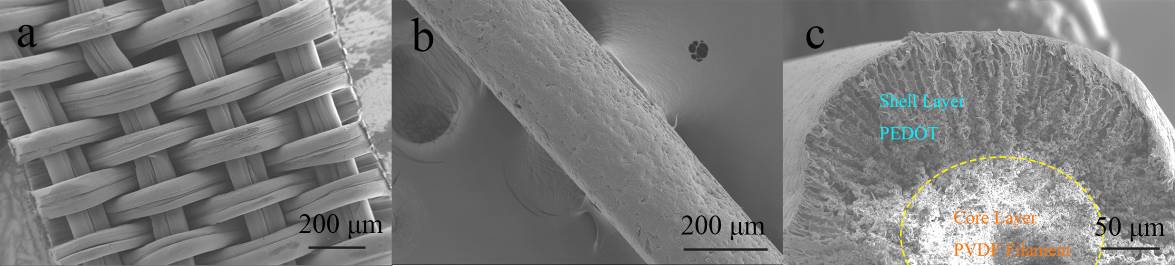


Figure S7 SEM images of (a) a common PEDOT-coated PVDF filament fabric. (b) Surface and (c) cross section of a PEDOT-coated PVDF filament.


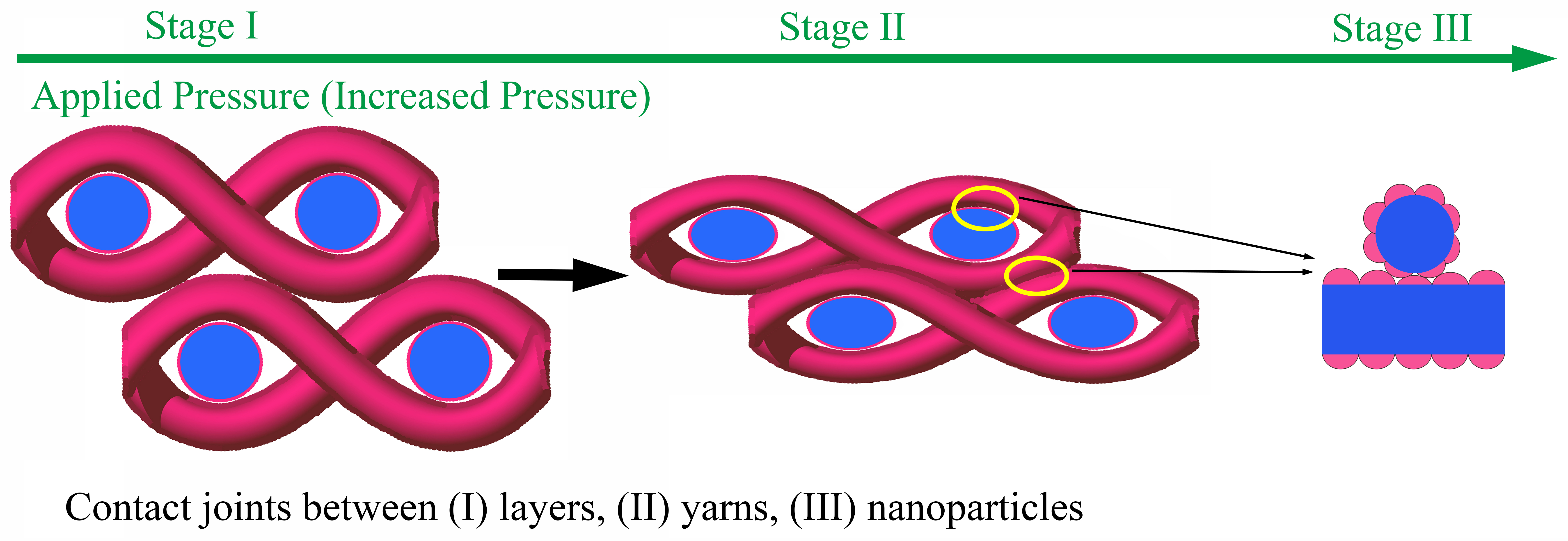


Figure S8 Schematic diagram of the main structural change in PEDOT-coated PVDF filament woven fabric sensor during the compression process.


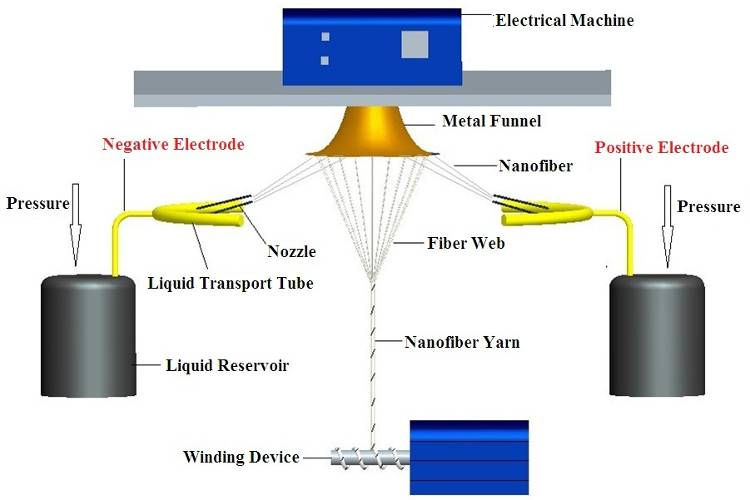


Figure S9 Schematic of double-conjugate electrospinning device for preparing nanofiber yarns.


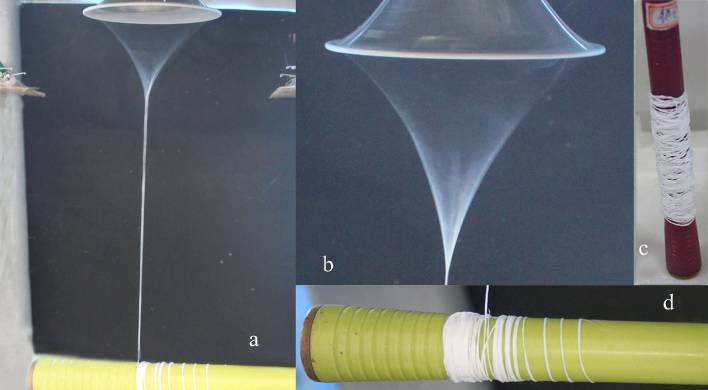


Figure S10 Experimental images of nanofiber yarn fabricated by double-conjugate electrospinning. (a) Schematic of the fabrication of continuous nanofiber yarn; (b) inverted cone-shaped hollow fiber web; (c) production of nanofiber yarn; and (d) winded nanofiber yarn.
